# Characterization of beta-tubulin DNA sequences within *Candida parapsilosis* complex

**DOI:** 10.18502/cmm.4.1.31

**Published:** 2018-03

**Authors:** Mahboobeh Kharazi, Bahram Ahmadi, Koichi Makimura, Armin Farhang, Sahar Kianipour, Marjan Motamedi, Hossein Mirhendi

**Affiliations:** 1 Department of Medical Parasitology and Mycology, International Campus, Tehran University of Medical Sciences, Tehran, Iran; 2 Department of Medical Laboratory Sciences, School of Para-Medicine, Bushehr University of Medical Sciences, Bushehr, Iran; 3 Laboratory of Space and Environmental Medicine, Graduate School of Medicine, Teikyo University, Tokyo, Japan; 4 Department of Medical Parasitology and Mycology, School of Medicine, Isfahan University of Medical Sciences, Isfahan, Iran; 5 Department of Medical Parasitology and Mycology, School of Medicine, Shiraz University of Medical Sciences, Shiraz, Iran

**Keywords:** Beta-tubulin, *Candida parapsilosis*, Sequence analysis

## Abstract

**Background and Purpose:**

*Candida parapsilosis* is a common cause of candidemia in children and patients with onco-hematological diseases, septic arthritis, peritonitis, vaginitis, and nail and skin infections. Regarding this, the present study was condcuted to evaluate intra- and inter-species variation within beta-tubulin DNA sequence of *C. parapsilosis* complex in order to establish the utilization of this gene in the identification and phylogenetic analysis of the species.

**Materials and Methods:**

A total of 23 isolates representing three different species of *C. parapsilosis* complex were used in this study, all of which were identifed by ITS-sequencing. For the successful amplification of beta-tubulin gene, a newly designed set of pan-*Candida* primers was used, followed by bilaterally sequence analysis for pairwise comparisons, determination of multiple alignments, evaluation of sequence identity levels, counting sequence difference, and construction of phylogenetic tree.

**Results:**

The multiple alignment of 623-629 bp-long nucleotide (nt) sequences reflecting the beta-tubulin gene indicated an inter-species divergence ranging within 0-68 nt in *C. parapsilosis*, *C. orthopsilosis*, and *C. metapsilosis* with a mean similarity of 84.7% among the species. Meanwhile, the intra-species differences of 0-20 and 0-6 nt were found between the strains of *C. parapsilosis* and *C. orthopsilosis*, respectively. The phylogenetic tree topology was characterized by a clade made up by *C. parapsilosis* and *C. orthopsilosis*, while *C. metapsilosis* formed a related but separate lineage.

**Conclusion:**

Our data provided the basis for further discoveries of the relationship between the species belonging to *C. parapsilosis* complex. Furthermore, the findigns of the prsent study revealed the efficiency of beta-tubulin DNA sequence data in the identification and taxonomy of *C. parapsilosis* and other pathogenic yeasts.

## Introduction

*Candida parapsilosis* is a common commensal of the skin that can cause candidemia in children and onco-hematologic patients, due to its ability to adhere to vascular catheters, prosthetics devices, and the skin of health care workers [1-3]. This species can also affect the patients with septic arthritis, peritonitis, vaginitis, as well as nail and skin infections [4, 5]. Early reports showed that *C. parapsilosis* is genetically more heterogeneous than other *Candida* species.

Based on molecular techniques, such as randomly ampliﬁed polymorphic DNA (RAPD), DNA sequencing, and morphotyping [6, 7], this species is divided into three groups, including *C. parapsilosis* I, II, and III [8]. However, molecular fingerprinting and mitochondrial genome signatures have shown that these groups are related to three different species, namely *C. parapsilosis *sensu stricto, *C. orthopsilosis*, and *C. metapsilosis* [9, 10]. 

The use of phylogenetic species concepts based on ribosomal DNA regions has greatly improved the taxonomy of yeasts. According to ISHAM-ITS reference database (http://its.mycologylab.org/), there is an intra-species diversity in *C. parapsilosis* complex. Nevertheless, confirmation and refinement using other genes is long overdue. The description and characterization of new genetic markers for *C. parapsilosis* complex can clarify its taxonomy and might be helpful for detection/identification purposes. 

The protein coding genes, such as beta-tubulin (*BT2*), have been proven to be a powerful tool for the species delimitation of the closely related species, which have been successfully used for the species delineation of fungal groups, such as *Aspergillus* [11], *Penicillium* [12], *Scedosporium* [13], *dermatophytes* [14, 15], and *Phaeoacremonium* [16]. With this background in mind, the present study was conducted to compare *BT2* gene sequences with ITS sequences within the *C. parapsilosis* complex and investigate its resolution power as a new genetic marker with regard to intra- and inter-species variation and application in phylogenetic analysis, taxonomy, and identification.

## Materials and Methods

A total of 23 isolates representing three different species of *C. parapsilosis* complex, including 20 clinical isolates, 2 ATCC, and 1 TIMM reference strains (Table 1), were subjected to *BT2* gene sequencing. The clinical isolates were selected from a collection of strains isolated from blood and other normally sterile clinical samples, which had been already collected from the children admitted to the Pediatric Inensive Care Unit of the Pediatric Medical Centers of Tehran, Iran [17].

DNA extraction and purification from the yeast colonies was accomplished using a previously described method [18]. For the preliminary identification of the strains, the ITS1-5.8SrDNA-ITS2 region was amplified using ITS1 (5′-TCC GTA GGT GAA CCT GCG G-3′) and ITS4 (5′-TCC TCC GCT TAT TGA TAT GC-3′) primers. The polymerase chain reaction (PCR) products were subjected to digestion with a restriction enzyme, namely *Msp*I (Fermentas, Vilnius, Lithuania), as previously described [19]. To confirm identification, all PCR products were subjected to sequencing of the entire ITS region using both ITS1 and ITS4 primers, and the sequences were compared with valid reference sequences deposited in the GenBank by Blast (https://blast.ncbi.nlm.nih.gov/Blast).

For the sequence analysis of *BT2* gene, the *BT2* sequences of various fungal species were obtained from the GenBank and aligned using the Geneious software (http://www.geneious.com). A novel set of pan-*Candida* primers was designed and named as: BCF (5’-AAG AAT TCC CTG ATA GAA TGA TG-3’) and BCR (5’-CCA ATG CAA GAA AGC TTT TCT T-3’). The PCR reactions contained 12.5 μL of premix (Ampliqon, Denmark), 2 μL (around 1 ng) of DNA template, 0.5 μM of each primers, and enough water up to a final reaction volume of 25 μl. The reaction mixture was initially denatured at 95°C for 5 min, followed by 35 cycles of 30 sec at 94°C , 45 sec at 55°C, and 45 sec at 72°C, and a terminal extension step of 72°C for 5 min. 

For the strains that failed to amplify, a nested PCR was set up for the successful amplification of the gene using BCFN (5’-AAG AAT TCC CTG ATA GAA TGA TG-3’) and BCRN (5’-CCA ATG CAA GAA AGC TTT TCT T-3’) primers. Subsequently, 1 μL of the 1:50-diluted product of the first PCR was added as a template to the reaction mixture of the second PCR and subjected to the above-mentioned thermal conditions. 

The PCR products were purified and sequenced bilaterally with the BCFN and BCRN primers using the ABI PRISM BigDye Terminator Cycle Sequencing Ready Reaction Kit (Applied Biosystems, Foster City, CA, USA) on an automated DNA sequencer (ABI PrismTM 3730 Genetic Analyzer, Applied Biosystems) according to the manufacturer’s instructions. 

Forward and reverse sequences of each sample were subjected to ClustalW pairwise alignment using Geneious and MEGA6 software [20]. Furthermore, the consensus sequences were entered into BioEdit software, version 7.0.5 [21] for the determination of multiple alignments, evaluation of sequence identity levels, counting of sequence difference, and construction of phylogenetic tree.

A phylogenetic tree was built using the maximum-likelihood algorithm with the Tamura-Nei parameter as a substitution model in MEGA6. The reliability of the branches was assessed using the bootstrap method with 1,000 simulations. The nucleotide sequences obtained in the study and their corresponding amino acid sequences were deposited in the GenBank, under the accession numbers of MH352134 to MH352145. 

**Table 1:** Reference and clinical strains of *C. parapsilosis* complex used for the analysis of partial beta-tubulin genes

**Species (total number of tested strains)**	**Strains**	**Length of Beta-tubulin DNA sequence (bp)**	**Range of intra-species variation in nucleotides**	**Mean similarity within the species (%)**
*Candida. parapsilosis* (15)	ATCC 90018, TIMM 2209, Ci 97, Ci 105, Ci 110, Ci 125, Ci 194, Ci 196, Ci 200, Ci 229, Ci 241, Ci 254, Ci 265, Ci 318, Ci 363	623-629	0-20	96.5
*Candida. orthopsilosis* (7)	Ci 168, Ci 187, Ci 378, Ci 423, Ci 423O, Ci 534, Ci 606	623-627	0-6	98.7
*Candida. metapsilosis* (1)	ATCC 96144	625	-	-

Ci: clinical isolate; ATCC: American type culture collection, TIMM: Teikyo University Institute of Medical Mycology

## Results

**Figure 1:**
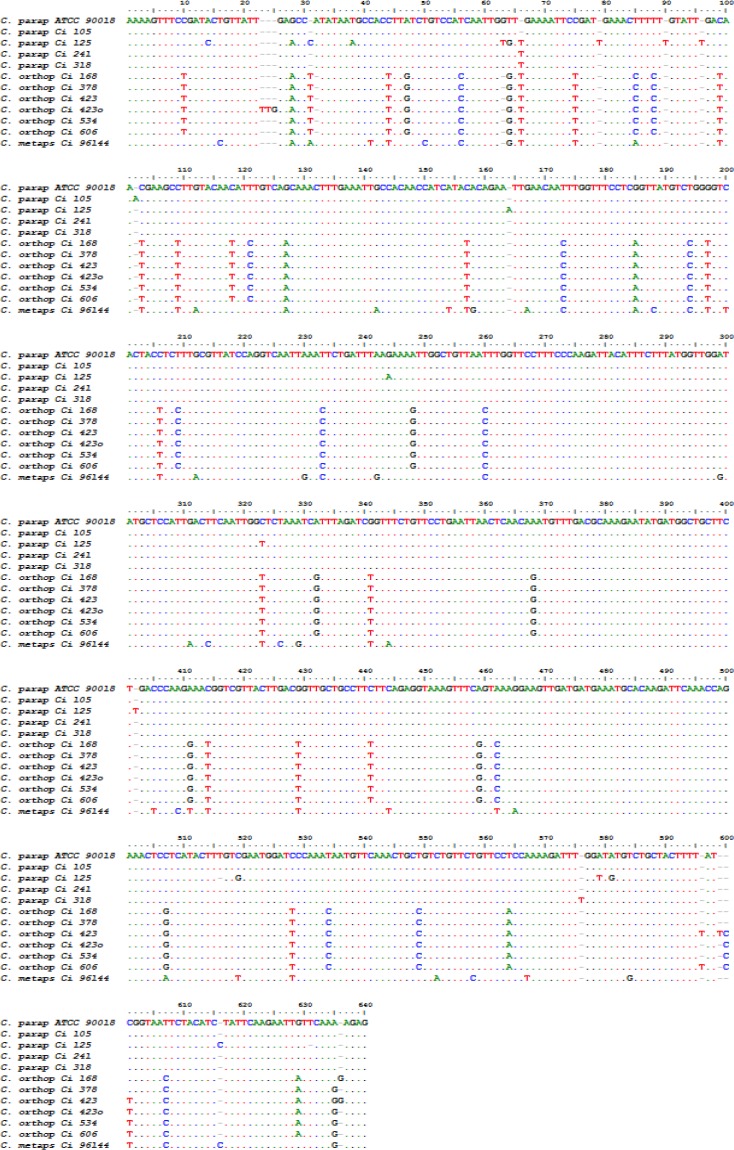
Multiple sequence alignment of partial *BT2* gene DNA sequences of strains belonging to *C. parapsilosis *complex (A dot indicates an identical nucleotide with respect to the top sequence; a dash indicates an insertion/deletion (indel) event.)

The primers designed in this study successfully amplified the target with a single band for all tested strains. Sequence analysis by BioEdit showed inter-species polymorphism ranging within 623-629 nucleotides (nt). The multiple alignment of the sequences indicated a mean similarity of 84.7% between the species. The sequence difference count matrix created by BioEdit showed significant differences among the species belonging to *C. parapsilosis* complex, including insertions/deletions and substitutions within the complex indicating inter-species divergence ranging from 0-68 nt (Figure 1). 

The largest inter-species nucleotide difference was observed between *C. metapsilosis* (ATCC 96144) and *C. parapsilosis* clinical isolate 125 with 68 nt. Meanwhile, intra-species differences were found within both *C. parapsilosis* and *C. orthopsilosis* by 0-20 and 0-6 nt, respectively (data not shown). The inter-species differences facilitated the distinction of five and six distinct *BT2* genotypes in *C. parapsilosis* and *C. orthopsilosis*, respectively. Because only one strain of *C. metapsilosis* was tested, we could not evaluate the intra-species variation within this species. Bioinformatic analysis and nucleotide BLAST search revealed no introns in the fragments, and it seemed that the region was evolutionarily conserved. 

**Figure 2:**
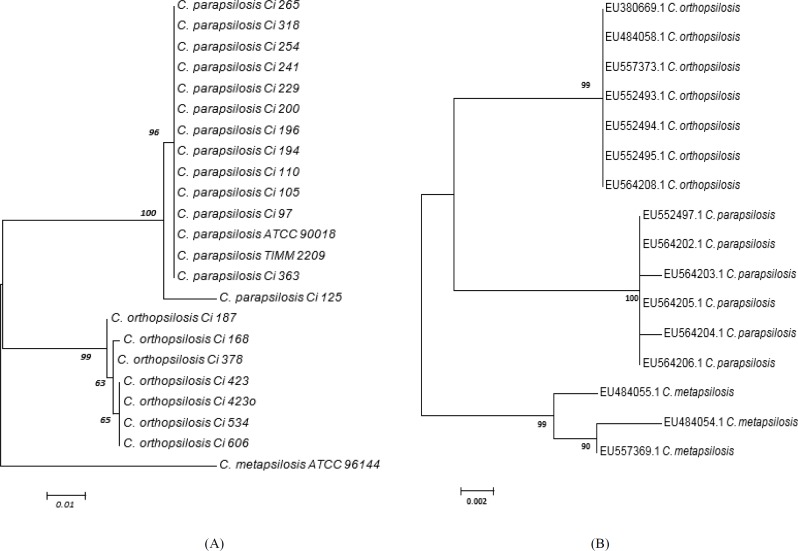
A) Phylogenetic analysis of *BT2* gene of 23 DNA isolates of *Candida parapsilosis* complex, B) phylogenetic analysis of 18S-ITS1-5.8S-ITS2-28S ribosomal RNA genes from representative *C. parapsilosis* strains, for which the data had been deposited in GenBank (evolutionary history was inferred using the maximum-likelihood method based on the Tamura–Nei model.)

Figure 2A illustrates the *BT2* gene tree topology as computed by the MEGA6 software. The backbone of the tree had high bootstrap values (70%) within the species of *C. parapsilosis* complex. Moreover, the interspecies correlations were obvious in the clades (Figure 2A). The *BT2* gene tree topology of the species was similar to that inferred from the ITS region analysis, with species clustering in similar strongly supported clades (Figure 2B). Sequence variation between the *Candida* strains led to the formation of a clade consisting of *C. parapsilosis* and *C. orthopsilosis*, while *C. metapsilosis* strain formed a separate lineage closely related to the clade consisting of *C. parapsilosis* and *C. orthopsilosis*.

## Discussion

*Candida parapsilosis* is the second most common yeast involved in bloodstream infections among neonates, catheter-associated candidemia, and intravenous hyperalimentation in different regions, such as Latin America, Asia, and Europe [22-24]. Highly variable (ITS1 and ITS2) and conserved (18S, 5.8S and 28S) regions of ribosomal DNA have been used for the detection and differentiation of medically important *Candida *species. However, the improvement of the current databases by the identification of new genetic markers establishes a foundation for the better distinction of the closely related species [25]. 

The sequence difference count matrix of *BT2* gene observed among the members of *C. parapsilosis *complex indicated that this locus may be more useful than ITS regions (84.7% versus 89.6% similarity) for the discrimination of these three closely related species (data not shown). Meanwhile, the analysis of the hyphal wall protein 1 (*HWP1*) nucleotide sequence alignment revealed only 60% similarity between *C. parapsilosis* and *C. orthopsilosis* [26]. 

*BT2* sequence variation between *C. parapsilosis* complex species ranged within 0-68 nt, which was similar to that of ITS sequences retrieved from the GenBank (0-67 nt). This finding was in line with the results obtained through the pyrosequencing of ITS2, sequencing of ITS1, and restriction fragment length polymorphism patterns of the intergenic spacer region 1 [8, 9, 27, 28].

Furthermore, the analysis of intein (in vacuolar *ATPase* gene, VMA) in 85 strains of *C. parapsilosis *complex showed that this locus is able to discriminate the members of *C. parapsilosis *complex based on VMA intein sizing. In this regard, *C. metapsilosis* exhibits a VMA intein smaller than that of *C. orthopsilosis* [29]. The intra-species DNA sequence variations of 0-20 and 0-6 nt led to the identification of five and six distinct *BT2* genotypes in *C. parapsilosis* and *C. orthopsilosis*, respectively. 

The association between distinct genetic variants and emergence of *C. parapsilosis* complex in various clinical settings has not been fully elucidated yet [30]. The presence of more intra-species sequence variation in *BT2* gene than in ribosomal genes makes this locus a potential candidate for the discrimination of organisms at the strain rather than the species level. Based on the in silico analysis of ITS1-5.8S-ITS2 region, *C. parapsilosis* and *C. orthopsilosis* had the intraspecific polymorphisms of 1-4 and 0-8 nt, respectively (data not shown). In addition, another analysis based on ISHAM-ITS reference database (http://its.mycologylab.org) revealed that *C. parapsilosis, C. orthopsilosis, *and* C. metapsilosis *had 2, 5, and 4 polymorphic sites, respectively.

Greater genetic variability of *C. orthopsilosis* in comparison to that of *C. parapsilosis* as observed by RAPD analysis has caused difficulties in the development of molecular techniques for the subtyping of these yeasts [9, 31]. The *BT2* gene tree topology constructed by means of the maximum-likelihood method revealed a cluster consisting of *C. parapsilosis* and *C. orthopsilosis*, with *C. metapsilosis* coming out as a separate lineage. 

The phylogeny of *C. parapsilosis* complex based on *BT2* gene is similar to the one inferred by using *HWP1* gene [26], D1/D2 region of ribosomal RNA gene [32], ITS, and 26S rRNA gene [33] sequences confirming the placement of *C. parapsilosis* and *C. orthopsilosis* in ’*psilosis*’ clade. 

## Conclusion

The present study is the first attempt targetted toward the evaluation of *BT2* gene as a new marker for the delineation of *C. parapsilosis* complex members. The obtained data can provide a basis for further discovery regarding the relationships of the closely related yeast species. The constructed tree topologies showed a high concordance between *BT2* and those observed for other markers, such as *HWP1*, D1/D2 region of 26SrDNA, and ITS1-ITS2. According to intraspeciﬁc polymorphism observed in *C. parapsilosis* and *C. orthopsilosis* species, it is needed to perform further studies on longer portions of *BT2* gene to test the potentiality of *BT2* to be used for genotyping. 

## References

[1] Levin A, Costa S, Mussi N, Basso M, Sinto S, Machado C (1998). Candida parapsilosis fungemia associated with implantable and semi-implantable central venous catheters and the hands of healthcare workers. Diagn Microbiol Infect Dis.

[2] Kuhn DM, Mukherjee PK, Clark TA, Pujol C, Chandra J, Hajjeh RA (2004). Candida parapsilosis characterization in an outbreak setting. Emerg Infect Dis.

[3] Arsic Arsenijevic V, Otasevic S, Janic D, Minic P, Matijasevic J, Medic D (2018). Candida bloodstream infections in Serbia: first multicentre report of a national prospective observational survey in intensive care units. Mycoses.

[4] Nosek J, Tomaska Lu, Rycovska A, Fukuhara H (2002). Mitochondrial telomeres as molecular markers for identification of the opportunistic yeast pathogen Candida parapsilosis. J Clin Microbiol.

[5] Trabasso P, Matsuzawa T, Fagnani R, Muraosa Y, Tominaga K, Resende MR (2015). Isolation and drug susceptibility of Candida parapsilosis sensu lato and other species of C parapsilosis complex from patients with blood stream infections and proposal of a novel LAMP identification method for the species. Mycopathologia.

[6] Kato M, Ozeki M, Kikuchi A, Kanbe T (2001). Phylogenetic relationship and mode of evolution of yeast DNA topoisomerase II gene in the pathogenic Candida species. Gene.

[7] Lehmann PF, Lin D, Lasker B (1992). Genotypic identification and characterization of species and strains within the genus Candida by using random amplified polymorphic DNA. J Clin Microbiol.

[8] Lin D, Wu LC, Rinaldi MG, Lehmann PF (1995). Three distinct genotypes within Candida parapsilosis from clinical sources. J Clin Microbiol.

[9] Tavanti A, Davidson AD, Gow NA, Maiden MC, Odds FC (2005). Candida orthopsilosis and Candida metapsilosis spp nov to replace Candida parapsilosis groups II and III. J Clin Microbiol.

[10] Rycovska A, Valach M, Tomaska L, Bolotin-Fukuhara M, Nosek J (2004). Linear versus circular mitochondrial genomes: intraspecies variability of mitochondrial genome architecture in Candida parapsilosis. Microbiology.

[11] Balajee SA, Kano R, Baddley JW, Moser SA, Marr KA, Alexander BD (2009). Molecular identification of Aspergillus species collected for the transplant-associated infection surveillance network. J Clin Microbiol.

[12] Serra R, Peterson SW (2007). Penicillium astrolabium and Penicillium neocrassum, two new species isolated from grapes and their phylogenetic placement in the P olsonii and P brevicompactum clade. Mycologia.

[13] Gilgado F, Cano J, Gene J, Guarro J (2005). Molecular phylogeny of the Pseudallescheria boydii species complex: proposal of two new species. J Clin Microbiol.

[14] Rezaei-Matehkolaei A, Mirhendi H, Makimura K, de Hoog GS, Satoh K, Najafzadeh MJ (2014). Nucleotide sequence analysis of beta tubulin gene in a wide range of dermatophytes. Med Mycol J.

[15] Mirhendi H, Motamedi M, Makimura K, Satoh K (2016). Development a diagnostic pan-dermatophyte TaqMan probe real time PCR assay based on beta tubulin gene. Mycoses.

[16] Mostert L, Groenewald JZ, Summerbell RC, Robert V, Sutton DA, Padhye AA (2005). Species of Phaeoacremonium associated with infections in humans and environmental reservoirs in infected woody plants. J Clin Microbiol.

[17] Charsizadeh A, Mirhendi H, Nikmanesh B, Eshaghi H, Makimura K (2018). Microbial epidemiology of candidaemia in neonatal and paediatric intensive care units at the Children's Medical Center, Tehran. Mycoses.

[18] Ahmadi B, Mirhendi H, Shidfar MR, Nouripour-Sisakht S, Jalalizand N, Geramishoar M (2015). A comparative study on morphological versus molecular identification of dermatophyte isolates. Med Mycol J.

[19] Mirhendi H, Makimura K, Khoramizadeh M, Yamaguchi H (2006). A one-enzyme PCR-RFLP assay for identification of six medically important Candida species. Nihon Ishinkin Gakkai Zasshi.

[20] Tamura K, Stecher G, Peterson D, Filipski A, Kumar S (2013). MEGA6: molecular evolutionary genetics analysis version 60. Mol Biol Evol.

[21] Hall TA (1999). BioEdit: a user-friendly biological sequence alignment editor and analysis program for Windows 95/98/NT. Nucl Acids Symp Ser.

[22] Pfaller MA, Jones RN, Doern GV, Fluit AC, Verhoef J, Sader HS (1999). International surveillance of blood stream infections due to Candida species in the European SENTRY Program: species distribution and antifungal susceptibility including the investigational triazole and echinocandin agents. Diagn Microbiol Infect Dis.

[23] Pfaller MA, Jones RN, Doern GV, Sader HS, Messer SA, Houston A (2000). Bloodstream infections due to Candida species: SENTRY antimicrobial surveillance program in North America and Latin America, 1997-1998. Antimicrob Agents Chemo.

[24] Canela HMS, Cardoso B, Vitali LH, Coelho HC, Martinez R, Ferreira MEDS (2018). Prevalence, virulence factors and antifungal susceptibility of Candida spp isolated from bloodstream infections in a tertiary care hospital in Brazil. Mycoses.

[25] Alnuaimi AD, Wiesenfeld D, O'Brien-Simpson NM, Reynolds EC, Peng B, McCullough MJ (2014). The development and validation of a rapid genetic method for species identification and genotyping of medically important fungal pathogens using high-resolution melting curve analysis. Mol Oral Microbiol.

[26] Abastabar M, Hosseinpoor S, Hedayati MT, Shokohi T, Valadan R, Mirhendi H (2016). Hyphal wall protein 1 gene: A potential marker for the identification of different Candida species and phylogenetic analysis. Curr Med Mycol.

[27] Asadzadeh M, Ahmad S, Al-Sweih N, Khan ZU (2009). Rapid molecular differentiation and genotypic heterogeneity among Candida parapsilosis and Candida orthopsilosis strains isolated from clinical specimens in Kuwait. J Med Microbiol.

[28] Borman AM, Linton CJ, Oliver D, Palmer MD, Szekely A, Odds FC (2009). Pyrosequencing analysis of 20 nucleotides of internal transcribed spacer 2 discriminates Candida parapsilosis, Candida metapsilosis, and Candida orthopsilosis. J Clin Microbiol.

[29] Prandini TH, Theodoro RC, Bruder-Nascimento AC, Scheel CM, Bagagli E (2013). Analysis of inteins in the Candida parapsilosis complex for simple and accurate species identification. J Clin Microbiol.

[30] Dassanayake RS, Samaranayake LP (2000). Characterization of the genetic diversity in superficial and systemic human isolates of Candida parapsilosis by randomly amplified polymorphic DNA (RAPD). Apmis.

[31] Lasker BA, Butler G, Lott TJ (2006). Molecular genotyping of Candida parapsilosis group I clinical isolates by analysis of polymorphic microsatellite markers. J Clin Microbiol.

[32] Herkert PF, Gomes RR, Muro MD, Pinheiro RL, Fornari G, Vicente VA (2015). In vitro susceptibility and molecular characterization of Candida spp from candidemic patients. Rev Iberoam Micol.

[33] Mirhendi H, Bruun B, Schonheyder HC, Christensen JJ, Fuursted K, Gahrn-Hansen B (2010). Molecular screening for Candida orthopsilosis and Candida metapsilosis among Danish Candida parapsilosis group blood culture isolates: proposal of a new RFLP profile for differentiation. J Med Microbiol.

